# Retraction to: Economic viability in free-range chicken production

**DOI:** 10.1093/tas/txac034

**Published:** 2022-05-02

**Authors:** Dian Lourençoni, Amelia Carvalho Faustino, Sílvia Helena Nogueira Turco, Otoniel Cajuí Bonfim, Luana Carolina, Rocha E Silva, Ana Carolina De Sá Silva Lins

**Affiliations:** Agricultural and Environmental Engineering, Universidade Federal do Vale do São Francisco, Rua Antonio Carlos Magalhães, 510, Country Club, Juazeiro 48902-300, Brazil

Dian Lourençoni, Amelia Carvalho Faustino, Sílvia Helena Nogueira Turco, Otoniel Cajuí Bonfim, Luana Carolina Rocha E Silva, Ana Carolina De Sá Silva Lins, Economic viability in free-range chicken production, *Translational Animal Science*, 2021; txab109, https://doi.org/10.1093/tas/txab109

This article has been withdrawn by the authors following a stalemate during the article’s production process about the copyright license and has therefore been retracted.

